# Oxide Layer Formation on AA2024-T3 Aircraft Alloy via Anodization in Environmentally Conscious Organic Acid Electrolytes

**DOI:** 10.3390/ma19071291

**Published:** 2026-03-24

**Authors:** Christian Girginov, İrem Nisa Erçel, Stephan Kozhukharov, Aleksandar Tsanev, Ognian Dimitrov, Mariya Georgieva, Pavlina Bancheva-Koleva, Ani Stoilova, Plamen Petkov

**Affiliations:** 1 Department of Physical Chemistry, University of Chemical Technology and Metallurgy, 8 Kliment Ohridski Blvd., 1797 Sofia, Bulgaria; girginov@uctm.edu; 2 Laboratory of High Temperature Materials, Department of Metallurgy and Materials Engineering, Faculty of Engineering, Kocaeli University, Umuttepe, 41001 Kocaeli, Türkiye; iremnisaercel@gmail.com; 3 LAMAR Laboratory for Advanced Materials Research, University of Chemical Technology and Metallurgy, 8 Kliment Ohridski Blvd., 1797 Sofia, Bulgaria; bancheva@uctm.edu (P.B.-K.); ani_stoilova@uctm.edu (A.S.); p.petkov@uctm.edu (P.P.); 4 Department of Physics, University of Chemical Technology and Metallurgy, 8 Kliment Ohridski Blvd., 1797 Sofia, Bulgaria; 5 Institute of General and Inorganic Chemistry, Bulgarian Academy of Sciences, Acad. G. Bonchev Str., Bld. 11, 1113 Sofia, Bulgaria; tsanew@abv.bg; 6 Institute of Electrochemistry and Energy Systems, Bulgarian Academy of Sciences, Acad. G. Bonchev Str., Bld. 10, 1113 Sofia, Bulgaria; ognian.dimitrov@iees.bas.bg; 7 Institute of Mineralogy and Crystallography, Bulgarian Academy of Sciences, Acad. G. Bonchev Str., Bld. 107, 1113 Sofia, Bulgaria; mgeorgieva@imc.bas.bg; 8 Institute of Optical Materials and Technologies, Bulgarian Academy of Sciences, Acad. G. Bonchev Str., Bld. 109, 1113 Sofia, Bulgaria

**Keywords:** AA2024-T3 alloy, anodic polarization, environmental electrolytes, layer characteristics, barrier properties

## Abstract

The recent endeavor to establish a sustainable society, with respect to environmental protection and occupational health prevention, imposes the need for the development of environmentally friendly anodization electrolytes. In addition, these electrolytes should be composed of biocompatible organic acids derived from renewable sources. In response to these challenges, a need arises to seek environmentally conscious alternatives to the widely used sulfuric acid anodization electrolytes. Accordingly, a comparative study was performed on the anodic polarization of AA2024-T3 aircraft alloy samples for 30 min at 0 or 20 °C. The respective electrolytes were composed of 0.5 M solutions of oxalic, citric, tartaric acids, or glycine. The comparative analysis included optical metallographic microscopy (OMM), scanning electron microscopy (SEM), determination of color characteristics and wettability, chemical composition analysis by X-ray photoelectron spectroscopy (XPS), and assessment of the corrosion protective properties of the obtained layers. The latter were defined by the application of electrochemical impedance spectroscopy (EIS) and potentiodynamic polarization scanning (PDS) after 24 h of exposure to a 0.5% NaCl solution. Among the most important conclusions is that the barrier properties of the layers obtained in citric and tartaric acid electrolytes remarkably exceed those of the film obtained in oxalic acid. The use of glycine does not result in film formation at all. The process temperature did not have as strong an effect as the electrolyte composition.

## 1. Introduction

Aluminum has the inherent ability to re-passivate through the spontaneous formation of oxide layers. However, these layers exhibit amphoteric behavior, being readily soluble in non-neutral aqueous media and in the presence of Cl^−^ ions. This drawback is further exacerbated in strengthened structural Al-based alloys because intermetallic inclusions disrupt the integrity of the oxide layer and promote galvanic corrosion, which may evolve into other types of localized corrosion [1,2,3,4].

In particular, copper-based intermetallic inclusions in the AA2024-T3 aircraft alloy act as cathodic sites for the oxygen reduction reaction, promoting anodic dissolution of the main Al-matrix grains [5,6,7,8,9] and other, more active intermetallic inclusions [10,11,12,13,14,15].

A principal response to this disadvantage is the electrochemical thickening of the oxide layer by anodizing. This method also offers an additional advantage, enabling the formation of highly textured surfaces [16,17,18,19,20], providing opportunities for elaboration of surfaces with bactericide [21,22,23,24,25] and superhydrophobic [26,27,28,29,30,31,32,33,34,35] properties, together with the evidently prominent corrosion protective [36,37,38,39,40,41,42] and self-lubricating [43,44] abilities. In addition, recently aluminum anodization was used for the elaboration of oxide layers, suitable for optical devices [45,46,47] and sensor elements [48,49,50,51,52,53,54,55]. In this regard, the influence of various anodization parameters on the resulting oxide layer topology has been studied, including substrate pretreatment [56,57], composition [58], applied current density [59], voltage [60,61,62], anodization regime [63,64], and, of course, process duration [61,65,66,67]. Furthermore, special attention has been paid to the influence of additives in sulfuric acid electrolytes, such as citric acid [65] or ethylene glycol, boric acid, and/or AlCl_3_ [68].

It has been demonstrated that the anodic aluminum oxide (AAO) films obtained are suitable for efficient corrosion protection [69,70,71], the fabrication of energy converters [72], aluminum-air batteries [73], sensor elements [47,52,74,75], microelectronic components [58,76,77,78,79,80,81,82], catalyst supports for chemical synthesis and environmental applications [83,84,85], membrane technologies [51,86,87], and other important applications [88,89,90,91]. In this context, anodizing in sulfuric acid has become a common procedure for surface treatment of various Al- [92,93,94,95], Mg- [96,97], Zn- [98,99], and Ti-based materials [100,101].

Nevertheless, recent trends toward a sustainable society necessitate developing environmentally conscious alternatives to the commonly used anodization electrolyte [102,103,104].

For instance, oxalic acid is a common compound found in a variety of edible plants [105,106,107,108]. Its antinutrient effect, described elsewhere [109,110], is incomparably weaker than the toxic and tissue-damaging effect of H_2_SO_4_. Moreover, biocompatible technologies for its production have recently been proposed [111].

In turn, citric acid is recognized as a valuable commercial product used in the food industry and in the pharmaceutical, biomedical, textile, and leather industries [112,113]. Its use as a detergent, disinfectant, and extractant and for food preservation and environmental remediation is described by Ciriminna et al. [114]. Biotechnological methods for the industrial-scale production of this acid are also available [115,116,117,118,119].

Tartaric acid is a major component of grapes [120] and wine [121,122,123]. It is a conventional food additive, designated as E-334 by the *Scientific Committee for Food* [124], and possesses beneficial antioxidant properties [125]. Furthermore, biotechnological production of this compound from renewable sources is also possible [126,127].

Finally, glycine appears as the simplest amino acid. It is composed of proteins and thus forms the basis of all living organisms on Earth. Consequently, there is no need to demonstrate its biocompatibility. This compound can also be obtained by biotechnological methods on an industrial scale [128,129,130].

The combination of the benefits of the anodization process and the above-described oxalic, citric, and tartaric acids, as well as glycine, has motivated interest in their potential use in this process, applied at two different temperatures as a technological parameter.

Hence, the present research aims to conduct a comparative analysis of the use of these four natural organic acids as potential alternatives to H_2_SO_4_ as an anodization electrolyte. The comparative analysis included determining the topologies, surface properties, chemical compositions, and corrosion resistance of the obtained layers.

## 2. Experimental

### 2.1. Electrochemical Oxide Layer Deposition Procedures

Overall, 18 AA2024-T3 aircraft alloy plates were used in the present systematic study. All plates were subjected to preliminary treatments before anodizing to remove surface contaminants, including temporary corrosion-protective layers. The preliminary treatments consisted of etching (i.e., desmutting) for 2 min in a 50 g dm^−3^ NaOH solution, followed by degreasing in diluted (1:1 *v*/*v*) HNO_3_ at room temperature.

After the preliminary surface treatment, 16 samples were subjected to subsequent anodizing via anodic polarization under galvanostatic conditions at a current density of 15 mA cm^−2^ for 30 min at either 0 °C (8 samples) or 20 °C (8 samples). These temperatures were maintained using a JULABO FP 40 thermostat, product of JULABO GmbH, Seelbach, Germany. The remaining 2 samples served as references. Attempts to perform anodic polarization at lower temperatures were also made. However, the solutions froze under these conditions, and therefore, no further experiments were carried out.

The electrochemical layer formation was performed in a two-electrode cell, mounted inside the above-mentioned thermostat. The electrodes were positioned vertically, with a distance of 1 cm between them, inside a glass vessel containing 400 mL of electrolyte. The counter electrode was a platinum mesh with a semi-cylindrical shape in order to ensure uniform distribution of the electric field over the entire surface area of the working electrode. The cell was additionally equipped with a thermometer and a compact stirrer, positioned near the electrodes. The above-described galvanostatic conditions were maintained using a GW Instek GPR-100H05D DC power supply, produced by Good Will Instrument Co. Ltd, Taipei, Taiwan. The current density was additionally monitored by a Mastech MS 8050 digital multimeter, product of the same manufacturer.

The anodization process was monitored with simultaneous data acquisition. Thus, the layer formation voltages (*U*_f_) were recorded to describe the process kinetics by means of in situ *U*_f_(*t*) curves. The data acquisition was performed using PeakTech 4000 digital multimeter, provided by Good Will Instrument Co. Ltd, New Taipei City, Taiwan. The output signals from these instruments were collected and analyzed using a commercial computer system.

Four environmentally friendly organic acid electrolytes were used in the present study. The first electrolyte was prepared by dissolving 63.4153 g of oxalic acid (C_2_H_2_O_4_·2H_2_O, 99.5 wt.%) to obtain a concentration of 0.5005 mol dm^−3^. The second electrolyte consisted of 106.1120 g of citric acid (C_6_H_8_O_7_·H_2_O, 99.8 wt.%), resulting in a concentration of 0.5040 mol dm^−3^. The third solution was composed of 0.5050 mol dm^−3^ tartaric acid (C_4_H_6_O_6_, dry, 99.5 wt.%), obtained by dissolving 38.0278 g of the dry compound. The final electrolyte was prepared by dissolving 75.3832 g of glycine (NH_2_CH_3_CO_2_, dry, 99.7 wt.%) to obtain a concentration of 0.5023 mol dm^−3^. All solutions were prepared in 1 dm^3^ of distilled water using volumetric flasks.

### 2.2. Topological Characterization of the Samples

Optical metallographic microscopy (OMM) images were acquired using an “Optika” microscope (Italy) at high magnification, with an “Optika PRO6” digital camera. Both provided by M.A.D. Apparecchiature Scientifiche srl, Bergamo, Italy.

Additionally, scanning electron microscopy (SEM) images were acquired using a Zeiss EVO 10 microscope (Carl Zeiss Microscopy, Oberkochen, Germany) in secondary electron mode at an accelerating voltage of 15 keV.

### 2.3. Surface Properties of the Studied Samples

#### 2.3.1. Color Characteristics

The color parameters of all samples were measured using a Lovibond RT 100 tintometer, product of Tintometer GmbH, Dortmund, Germany. Two measurements were performed on the front and back sides of each sample (i.e., four points per sample pair). The results were interpreted using the CIE L*a*b* color system.

#### 2.3.2. Wettability Tests

Contact angle measurements were performed using a “Theta Lite” high-precision optical device (Biolin Scientific, Gothenburg, Sweden, coupled with specialized “One Attention” software provided from the same company. A constant droplet volume was maintained using a “Gastight 1001” precision screw syringe (Hamilton Co., Reno, NV, USA). Measurements were taken at eight points on each sample (i.e., 16 points per sample pair).

#### 2.3.3. Chemical Composition of the Studied Samples

XPS analyses were performed using a VG Escalab II system product of Vacuum Scientific Ltd., East Grinstead, UK with Al Kα radiation at 1486.6 eV. The chamber pressure was 1 × 10^−9^ Torr. The adventitious carbon C1s line at 284.6 eV was used as an internal standard for binding energy calibration. The photoelectron spectra were corrected by subtracting a Shirley-type background and quantified using peak areas and Scofield’s photoionization cross-sections. The accuracy of the measured binding energies was ± 0.2 eV.

### 2.4. Electrochemical Testing Procedures

These procedures were performed after 24 h of exposure to the model corrosive medium, consisting of 0.5% NaCl solution.

#### 2.4.1. Electrochemical Impedance Spectroscopy

EIS spectra were acquired using an Autolab 30 PG-stat equipped with a frequency response analyzer (FRA-2), both produced by Metrohm, Autolab B.V., Utrecht, The Nedtherlands. Excitation signals were applied to the electrochemical cells via a cylindrical platinum mesh surrounding the reference electrode. Spectra were recorded with 50 data points over the frequency range from 10 kHz to 0.01 Hz, using a 10 mV excitation signal relative to the open-circuit potential (OCP). The OCP was measured immediately before each measurement. Additional experiments were performed with 3.5% NaCl, however this concentration appeared too aggressive and did not allow for comparative analyses. The working electrode area was defined by O-rings with an internal diameter of φ = 13.6 mm.

#### 2.4.2. Potentiodynamic Polarization Scanning

PDS curves were recorded over a potential range from −50 to +500 mV relative to the OCP at a potential sweep rate of 10 mV s^−1^ using the mentioned above device.

## 3. Results and Discussion

### 3.1. In Situ Layer Deposition Kinetic Curves

**Figure 1:**
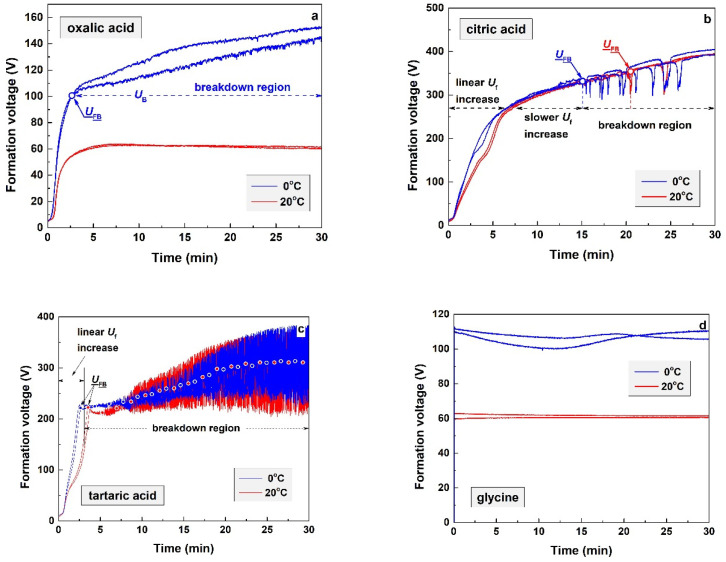
Juxtaposition between in situ kinetic curves recorded during electrochemical layer formation at 0 °C and 20 °C for: (**a**) oxalic acid; (**b**) citric acid; (**c**) tartaric acid and (**d**) glycine.

As already mentioned in Section 2 (*Experimental*), anodic aluminum oxide (AAO) layers were formed in four organic acid electrolytes at two temperatures (0 and 20 °C). The process was performed with simultaneous recording of the formation voltage (*U*_f_) as a function of time (*t*), and the resulting curves are presented in Figure 1. The *U*_f_(*t*) dependences were recorded for two samples in each of the four studied acids at both 0 and 20 °C. The acquired curves exhibit distinct shapes that are strongly dependent on the acid electrolyte used. Their analysis is based on the concepts of typical anodizing process kinetics in sulfuric acid electrolytes [131].

The kinetics of the layers formed in oxalic acid at 0 °C (Figure 1a) resemble the typical curves observed during anodizing of aluminum in this acid. The initial linear *U*_f_ increase terminates when the first breakdown voltage (*U*_FB_) is reached. At this point, continuous voltage oscillations appear. Within this breakdown region, the oscillations are accompanied by a retarded increase in *U*_f_. In contrast, the kinetic curves acquired at 20 °C exhibit a different shape. At this temperature, the *U*_f_(*t*) kinetic curves indicate the formation of porous AAO layers. Despite the evident differences between the kinetic curves obtained at the two temperatures, the formed AAO layers are probably porous in both cases. Indeed, anodizing in oxalic acid electrolytes results in the formation of porous AAO layers, where the pore diameter, depth, and distribution depend on the applied electrical regime, temperature, and process duration [132,133,134,135,136,137,138,139].

In turn, the kinetic curves obtained during anodization in citric acid at 0 and 20 °C are rather similar (Figure 1b). Consequently, temperature does not have a notable effect on the resulting films. The shapes of the *U*_f_(*t*) kinetic curves indicate the formation of a barrier film. However, unlike the curves described above for oxalic acid, an intermediate interval is observed between the initial linear section and the appearance of *U*_FB_. This interval is characterized by a deceleration of the *U*_f_ increase.

The kinetic curves obtained during anodization in tartaric acid exhibit rather peculiar behavior (Figure 1c). As with citric acid, temperature does not affect the curve shapes. The most distinctive features of the *U*_f_(*t*) curves in this case are the attainment of very high *U*_f_ values at the end of the process (up to approximately 380 V) and the remarkable amplitude of the registered oscillations (exceeding 250 V).

Finally, anodic polarization in glycine proceeds in a completely different manner (Figure 1d). On the one hand, it is strongly temperature-dependent. On the other hand, the recorded kinetic curves do not indicate the formation of any AAO layer.

In summary, the kinetic curves in the first three cases (Figure 1a–c) exhibit two distinct stages: (i) a linear increase in *U*_f_ with time due to the gradual growth of the oxide layer; and (ii) a deceleration in the *U*_f_ increase after *U*_FB_ is reached, accompanied by intense voltage oscillations. It should also be noted that temperature exerts a distinct influence only in the cases of anodizing in oxalic acid (Figure 1a) and glycine (Figure 1d).

### 3.2. Outlooks of the Investigated Samples

**Figure 2:**
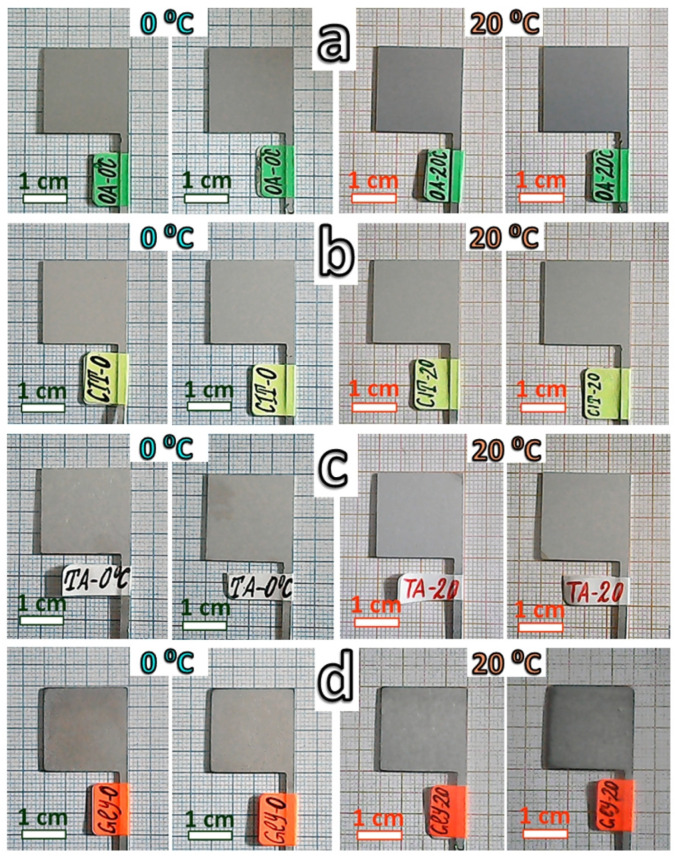
Photographs of layers formed at 0 and 20 °C in: (**a**) oxalic acid (OA); (**b**) citric acid (CIT); (**c**) tartaric acid (TA), and (**d**) glycine (Gly).

The appearances of the 16 investigated samples are shown in Figure 2.

Comparison of the photographs (Figure 2) reveals that the electrochemically deposited layers are uniform and completely cover the metallic surfaces. No metallic shine was observed, indicating that the layers are evidently dense. Oxalic acid and glycine result in the formation of darker layers, whereas citric and tartaric acids produce slightly brighter layers.

On the other hand, oxalic and citric acids yield relatively smoother surfaces than those obtained with the other two acids. The roughest surface, with evidently the lowest repeatability of the overall appearance, is observed for glycine. In this case, the surface layer is likely composed of agglomerates containing corrosion products.

### 3.3. Topological Observations

#### 3.3.1. Optical Metallographic Microscopy of the Investigated Samples

**Figure 3:**
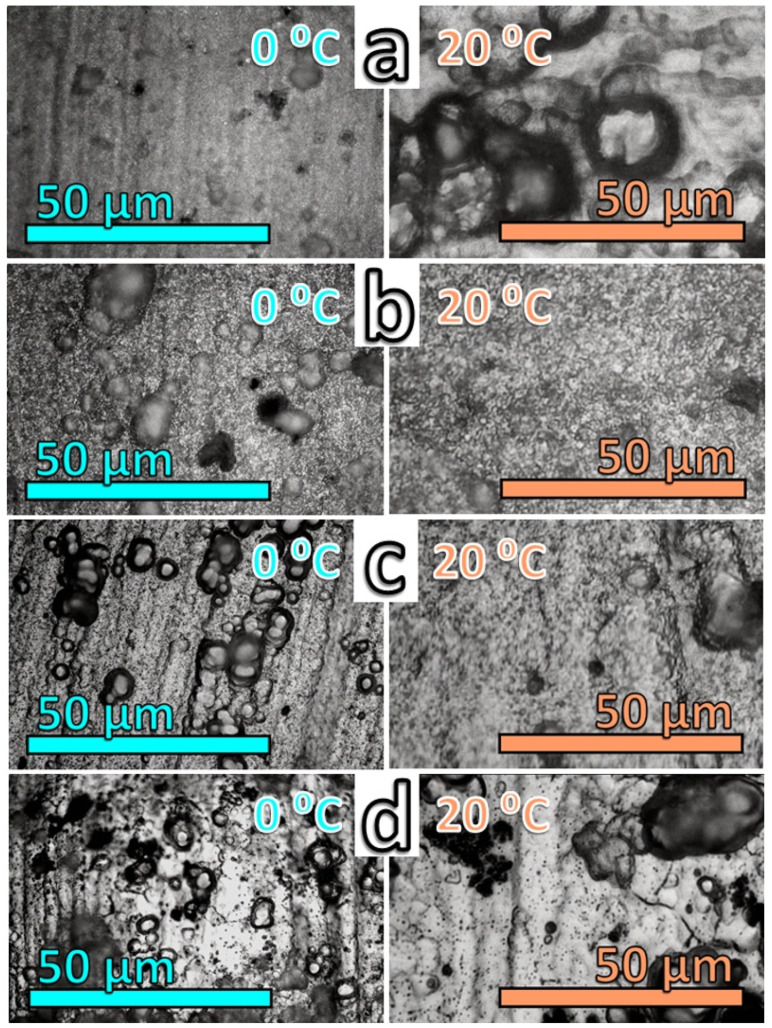
Optical metallographic microscopy (OMM) images of surfaces anodically polarized in: (**a**) oxalic acid; (**b**) citric acid; (**c**) tartaric acid, and (**d**) glycine.

The OMM images in Figure 3 reveal more distinct features of the layers formed in the respective acids. Comparison of the images indicates that the smoothest surfaces are those formed in oxalic acid at 0 °C, followed by those formed in citric acid at the same temperature. The other two acids exhibit a multitude of pits (tartaric acid) and larger dark zones (glycine).

The films formed at 20 °C have rougher surfaces than those formed at 0 °C for all investigated acids. In particular, for oxalic acid, the difference between the layers formed at 0 °C and 20 °C is rather remarkable.

As is well known from the literature [140,141,142], the pits observed in Figure 3 are likely formed during the preliminary treatments. However, anodic polarization in the respective acids may further increase the number and size of the particles. In the case of glycine, more aggressive conditions appear to be present, as the surfaces appear more severely affected after electrochemical treatment. This is because anodic polarization in this acid selectively dissolves the intermetallic inclusions, leading to pit and cavern formation. The electric current may also promote partial hydrolysis of glycine, forming ammonium ions that attack the AA2024 sample’s metallic surface.

#### 3.3.2. Scanning Electron Microscopy

**Figure 4:**
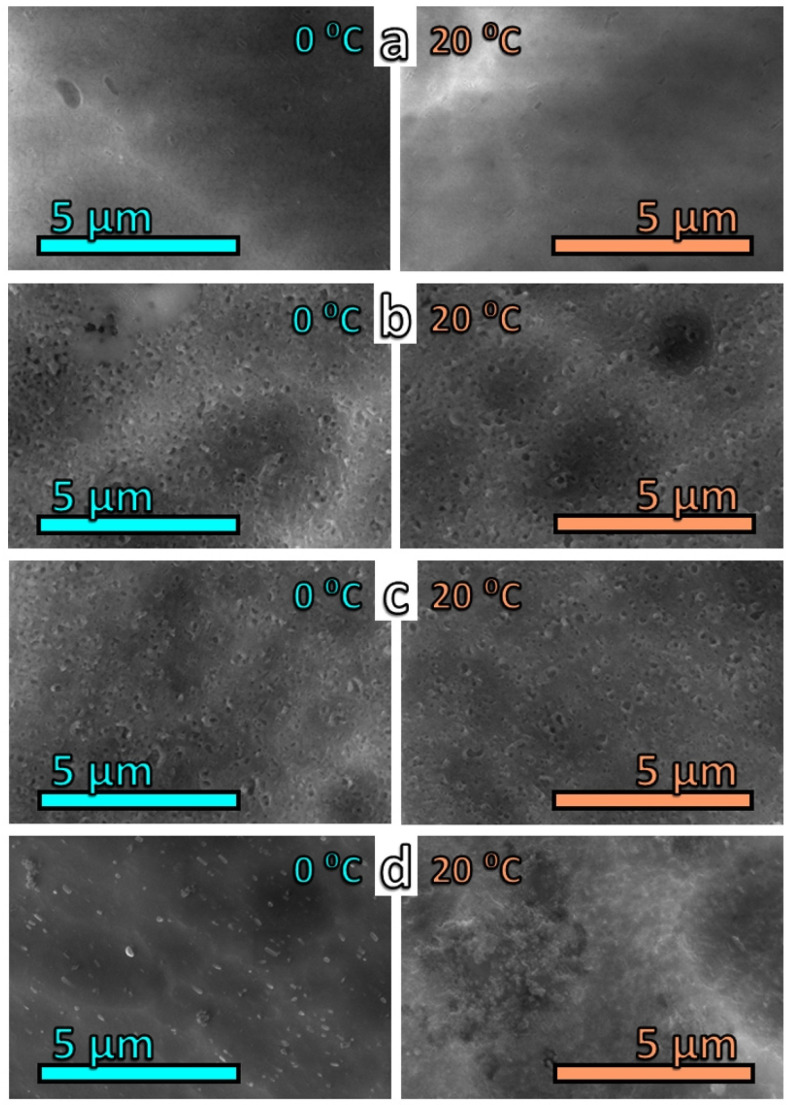
High-resolution topological SEM images of the layers electrochemically deposited at 0 and 20 °C in: (**a**) oxalic acid; (**b**) citric acid; (**c**) tartaric acid, and (**d**) glycine.

High-resolution SEM images show that oxalic acid forms a relatively thin, sparse, layered morphology (Figure 4a). This observation confirms the inference that oxalic-acid-derived AAO layers possess a porous morphology, as also reported by other authors [51,86,87,88,89,90,91,92].

Anodization in citric and tartaric acids yields well-defined layers with randomly distributed pores ranging from 50 to 250 nm in diameter (Figure 4b,c). Finally, the sample treated with glycine shows no continuous layer. Coarse intermetallic inclusions of the alloy are visible at 0 °C, whereas at 20 °C, a multitude of shapeless agglomerates appear instead of a uniform layer.

The surface topologies obtained by anodizing in the respective acids at 0 and 20 °C appear rather similar. The only notable difference between anodic polarization at 0 °C and 20 °C is observed for glycine. In the latter case, the metallic surface is covered by irregular agglomerates, as mentioned above. However, these aggregates cannot be considered AAO coatings.

### 3.4. Surface Properties of the Studied Samples

#### 3.4.1. Color Characteristics of the Investigated Samples

The color characteristics were determined using the CIE (L*a*b*) color system. The corresponding data for the samples anodized at 0 and 20 °C are summarized in Table 1.

The color characteristics of the reference samples were also determined. These specimens are distinctly bright, with insignificant color hues, possessing values of L* = 105.585 ± 1.739, a* = −0.325 ± 0.543, and b* = 0.930 ± 1.800.

After anodic polarization at 0 °C, the samples remain bright, as shown in Table 1. The only exception is observed samples polarized in oxalic acid, with L* ≈ 65. These samples exhibit slightly reddish (a* ≈ 2–3) and yellowish (b* ≈ 3–3.7) hues. The origin of these color tonalities is attributed to oxides and/or oxalates of the metals composing the intermetallic inclusions in the AA2024 alloy substrate.

The remaining samples exhibit more distinct color tonalities. According to the a* parameter, the samples treated with tartaric acid display a greenish hue (a* ≈ −2.63), whereas the others show negligible reddish coloration. The most pronounced yellow tonality is observed in samples polarized in citric acid (b* ≈ 10) and glycine (b* ≈ 7).

The anodizing at 20 °C results in layers with properties similar to those discussed above. Table 1 shows that all samples are bright (L* values above 90) and exhibit negligible reddish hues (a* below 1). Again, samples treated with oxalic acid show slightly more pronounced reddish coloration (a* between 1 and 5).

According to the b* color parameter, samples anodized in oxalic and citric acids exhibit more pronounced yellowish hues, with b* values exceeding 10. The remaining two acids (tartaric acid and glycine) show approximately twofold lower b* values.

#### 3.4.2. Surface Wettability of the Investigated Samples

Rather interesting results were obtained from the wettability tests. The AA2024-T3 alloy substrates after the initial surface treatments (i.e., the reference samples) exhibit a contact angle (*CA*) value of 64.61 ± 1.80°.

The corresponding *CA* values for the anodized samples are presented in Table 2. The analysis shows that the most hydrophilic layers are formed in oxalic acid, with CA ≈ 26° under both anodic polarization conditions at 0 and 20 °C.

Overall, the layers formed in citric acid are almost hydrophobic, with contact angles approaching *CA* → 80°. The most remarkable change in contact angle as a function of the layer formation temperature is observed for samples treated in tartaric acid, where *CA* increases from approximately 48° at 0 °C to about 67° at 20 °C. In the case of glycine, temperature has a negligible effect.

#### 3.4.3. Chemical Compositions of the Obtained Layers

The compositions of the surface layers were analyzed by X-ray photoelectron spectroscopy (XPS) to determine whether pure oxide layers were formed or whether organic acid anions were present in the layer. This analytical technique enabled the determination of both the elemental compositions and the chemical compounds that constitute the surface layers.

**Figure 5:**
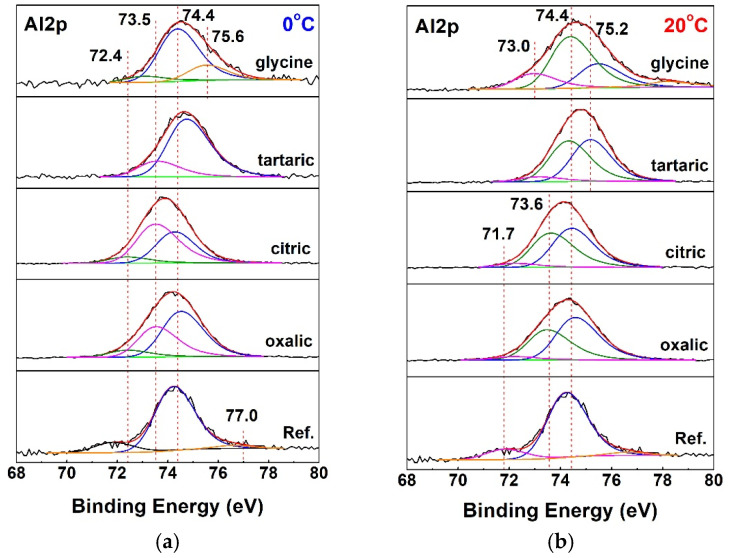
Peak-fitted XPS core-level Al2p spectra: (**a**) at 0 and (**b**) at 20 °C.

Peak-fitting analysis of the Al2p spectra (Figure 5) shows that the most intense peak for almost all samples is at 74.4 eV, indicating the presence of Al_2_O_3_ [143]. The only exception is the sample treated with citric acid at 0 °C. For glycine at both temperatures, a peak appears at 75.6 eV, revealing the presence of AlO(OH) [144]. This value is approximately 0.4 eV higher than the characteristic binding energy for this compound, which can be attributed to hydroxyl groups surrounding the Al cation. For tartaric acid, this peak shifts to a lower binding energy of 75.2 eV, typical of AlO(OH).

The spectra of samples anodized in citric and oxalic acids at 0 °C show additional low-intensity peaks at 72.4 eV, indicating the presence of metallic aluminum [145]. However, when the process is carried out at 20 °C, these peaks shift to lower binding energies, ranging from 71.7 to 71.9 eV, likely due to concomitant processes occurring on the sample surfaces during anodic polarization in the respective electrolytes.

The deconvoluted spectra of the anodized specimens also show peaks at approximately 73.5–73.6 eV. This binding energy range is characteristic of Al–O bonds within chelate complexes formed between aluminum and the respective organic acid anions.

**Figure 6:**
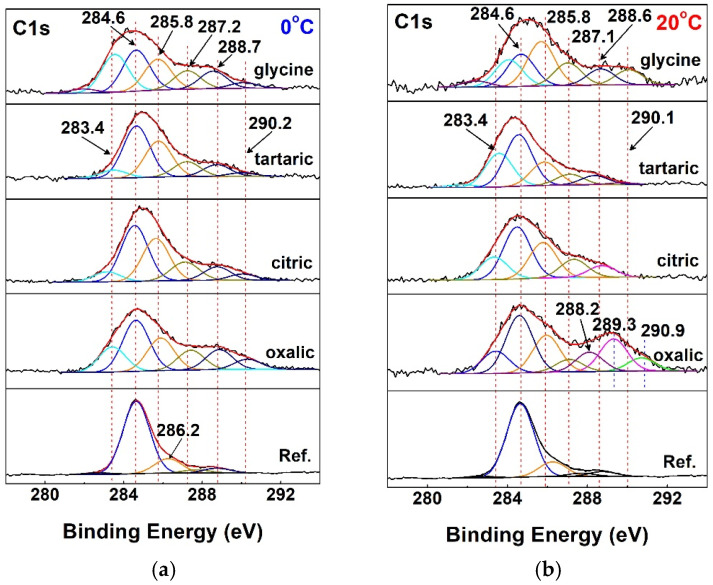
Peak-fitted XPS core-level C1s spectra: (**a**) at 0 and (**b**) at 20 °C.

Furthermore, the XPS spectra were calibrated using the C1s band at 284.6 eV, attributed to adventitious carbon and hydrocarbons (Figure 6). Peaks at 285.8 eV are characteristic of polar covalent bonds between carbon and hydroxyl moieties [146]. These OH groups originnate from aqueous electrolytes and from acid molecules, such as citric and tartaric acids. The number of OH groups does not depend on the anodization temperature at either 0 or 20 °C. The only exception is the sample treated in the glycine electrolyte at 20 °C.

In the reference sample, the peak at 286.2 eV arises from hydroxyl groups adsorbed during the preliminary treatments.

The occurrence of a peak at a C1s binding energy of 287.2 eV typically indicates the presence of carbonyl groups (C=O), which are often found in chelating ligands [147]. Peaks at 288.7 eV correspond to carboxyl groups [148].

During anodization in oxalic acid at 20 °C, the peak at 288.7 eV splits into two components at 288.2 and 289.3 eV, respectively. A possible reason for the shift in one peak by 0.5 eV toward lower binding energies is the formation of complexes between aluminum cations from the sample surface and oxalate anions from the electrolyte. In turn, the shift in the second peak by 0.5 eV toward higher binding energies indicates oxidation of oxalate to recently described crystalline metal–organic compounds [149] containing a novel C_4_O_7_^4−^ ligand, with compositions such as [(M^II^)_8_(C_4_O_7_)_4_(H_2_O)_12_]_24_·H_2_O.

Peaks in the binding energy range from 290.2 to 290.9 eV can be associated with bicarbonate species (HCO_3_)^−1^ and/or adsorbed or occluded CO_2_ produced by X-ray irradiation of oxalates [150]. Alternatively, this component may be attributed to plasmon satellites of “graphitic” C–C bonds [151,152].

A more detailed analysis of the C1s spectra shows the absence of organic acid moieties in the formed oxide layers. In this respect, the most representative C1s spectra are those of the samples treated with oxalic acid. Indeed, oxalic acid would be identifiable by the presence of a peak at 288.7 eV, corresponding to its carboxylic groups. However, such a peak is absent in the analyzed spectra, indicating the growth of a pure oxide layer during anodic polarization. More likely, the electrolytes used influence the structure and resulting topology of the layers, as evidenced by the smoother surfaces shown in Figure 4 (previous paragraph), in agreement with the observations of Tsanev et al. [153]. The highest Al_2_O_3_ content was observed in the samples treated with tartaric acid. This is accompanied by the presence of AlO(OH) in the case of this acid at 20 °C.

Samples anodically polarized in glycine exhibit the highest aluminum content, ranging from 34 to 43 at.%, compared with approximately 30 at.% in the other samples. This finding indicates that the film formed in the glycine electrolyte is the thinnest and/or most porous among the investigated samples. In addition, nearly 9 _at._% of metallic Al^0^ was detected on the surfaces of samples treated in oxalic- and citric-acid-based electrolytes.

**Figure 7:**
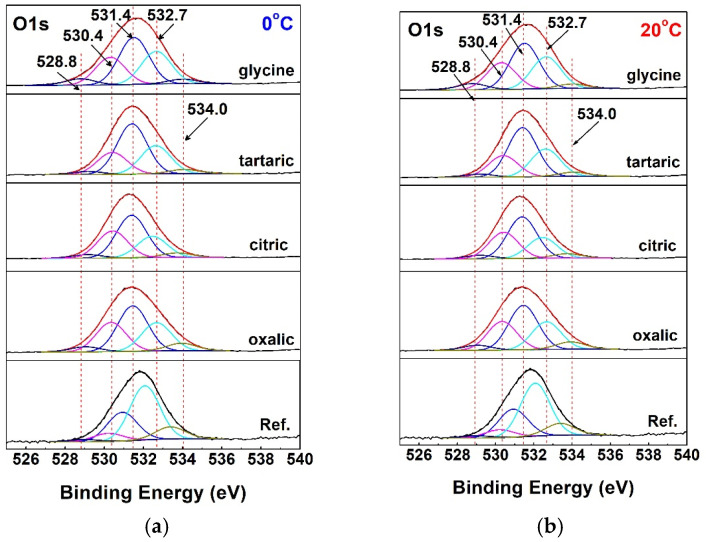
Peak-fitted XPS core-level O1s spectra: (**a**) at 0 and (**b**) at 20 °C.

Regardless of the acid used and the process temperature, all O1s core-level photoelectron spectra consist of five distinguishable peaks. For comparison, the peaks in the reference samples are shifted towards lower binding energy values because these specimens did not undergo anodic polarization (Figure 7).

The peak at 528.8 eV corresponds to lattice oxygen in metallic aluminum, also known as bulk oxygen. This low binding energy indicates that oxygen is strongly bonded to metal atoms in the bulk material [154]. The peak at 530.4 eV most likely corresponds to C–O–Al bonds. This lower-binding-energy peak may also indicate oxygen depletion around Al^3+^ ions [155,156].

The most pronounced peak, at 531.4 eV, arises from O–Al bonds in Al_2_O_3_. This peak is fully consistent with O–Al bonding in Al_2_O_3_, as reported in the literature [157,158].

The peak at 532.6 eV is attributed to the presence of C=O and COOH groups [159,160], whereas the peak at 533.9 eV corresponds to OOH groups (as in boehmite) and/or adsorbed or physically adsorbed water (H_2_O) [161].

### 3.5. Electrochemical Characterizations of the Investigated Samples

#### 3.5.1. Electrochemical Impedance Spectroscopy

**Figure 8:**
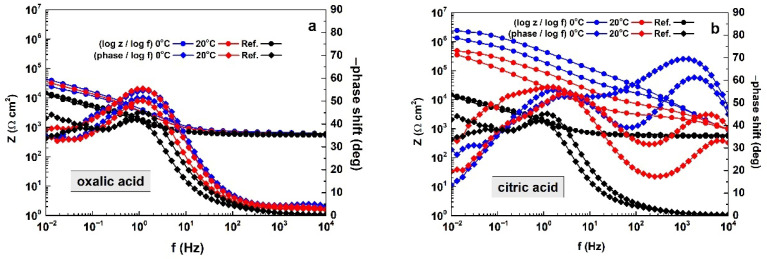

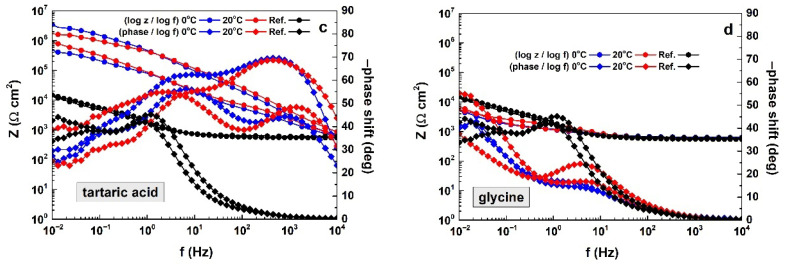
Juxtaposition among Bode plots of the electrochemical impedance spectra of the studied specimens, including the reference samples for: (**a**) oxalic acid, (**b**) citric acid, (**c**) tartaric acid and (**d**) glycine.

**Figure 9:**
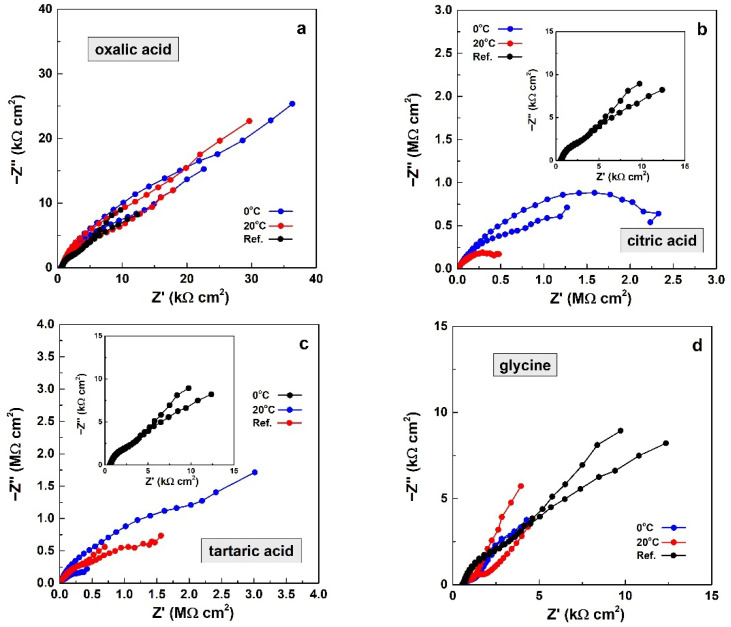
Juxtaposition among Nyquist plots of the electrochemical impedance spectra of the studied samples, including the reference samples, for: (**a**) oxalic acid, (**b**) citric acid, (**c**) tartaric acid and (**d**) for glycine.

This advanced instrumental method enables the determination of the barrier properties of layers formed during anodization in the respective acids (oxalic, citric, tartaric acids, and glycine) at 0 and 20 °C. The acquired spectra are shown in Figure 8 and Figure 9.

The Bode plots in Figure 8 reveal distinct differences in barrier properties among the obtained films, depending on the acid used for electrochemical layer formation. A key indicator of corrosion rate in the Bode plots is the total impedance at 0.01 Hz. For the reference samples, these values are |Z|0.01 Hz = 4.07 kΩ cm^2^ and |Z|0.01Hz = 3.63 kΩ cm^2^.

The spectra for oxalic acid (Figure 8a) resemble those of the reference samples. In this case, the impedance–frequency curves lie above those of the references, indicating the formation of distinct porous layers. According to the criterion mentioned above, the barrier ability of the films formed in oxalic acid at 0 °C slightly exceeds that of the layers formed at 20 °C. However, this difference is insignificant, since the former exhibit |Z|_0.01 Hz_ values in the range 26.92–44.25 kΩ cm^2^, whereas the latter reach |Z|_0.01 Hz_ of 21.48–37.33 kΩ cm^2^. Thus, the barrier properties (expressed by the impedance modulus at 0.01 Hz) of the layers formed in oxalic acid are approximately one order of magnitude higher than those of the reference samples.

The Bode plots for citric acid (Figure 8b) also show higher barrier properties at the lower temperature. For comparison, the total impedance of the films formed at 0 °C is |Z|_0.01 Hz_ = 1.44–2.30 MΩ cm^2^, whereas the films obtained at 20 °C exhibit |Z|_0.01 Hz_ values of 0.35–0.50 MΩ cm^2^. Moreover, the barrier properties achieved by anodizing in citric acid exceed those of the native oxide layers of the reference samples by up to three orders of magnitude.

In addition, the shapes of the spectra in this case clearly differ from those of the reference samples, as the phase angle–frequency plots show distinct pairs of peaks distributed across both the high- and low-frequency ranges.

The tartaric-acid-based electrolyte also yields films with significant barrier properties (Figure 8c). The layers formed at 0 °C exhibit |Z|_0.01 Hz_ values of 0.47–3.47 MΩ cm^2^, compared with |Z|_0.01 Hz_ values of 0.89–1.73 MΩ cm^2^ for the films obtained at 20 °C. Again, a lower temperature enables higher barrier performance, although the total impedance ranges overlap. Here, clearly distinguishable pairs of peaks appear in the phase angle–frequency curves, as in the previous case. This observation indicates that the capacitance of the formed layer is clearly distinguishable from that of the electric double layer. Consequently, the layers formed in citric and tartaric acids exhibit distinct barrier properties.

Finally, the use of glycine results in deterioration of the native oxide layers on the AA2024-T3 substrates, as evidenced by Bode plots that lie below those of the reference samples (Figure 8d). The total impedance values are nearly identical: |Z|_0.01 Hz_ = 5.17–5.70 kΩ cm^2^ at 0 °C and |Z|_0.01 Hz_ = 5.55–6.95 kΩ cm^2^ at 20 °C.

In summary, the Bode plots in Figure 8 indicate that films derived from oxalic acid exhibit relatively weak barrier properties. In contrast, anodizing in both citric and tartaric acids yields layers with substantially higher barrier properties. For oxalic, citric, and tartaric acids, the barrier properties of the layers formed at the lower temperature (0 °C) slightly exceed those of the films obtained at the higher temperature (20 °C). Anodic polarization in glycine at both temperatures has a pronounced detrimental effect overall.

The corresponding Nyquist plots (Figure 9) also reveal clear differences among the spectra. The plots recorded for oxalic acid (Figure 9a) are slightly larger than those of the reference samples and are nearly indistinguishable from one another. These plots display arcs with diffusion tails for all samples. Consequently, anodizing in oxalic acid forms layers with similar protective properties, regardless of the process temperature. Evidently, porous anodic aluminum oxide (AAO) layers are formed at both temperatures.

The spectra of films formed in citric acid (Figure 9b) consist of arcs with curved diffusion tails. The curvature indicates that these tails mark the initiation of additional arcs. Moreover, these spectra are significantly larger, reaching Z’ values of up to 2 MΩ cm^2^ and −Z″ values of up to 1 MΩ cm^2^ at 0 °C. The corresponding plots for layers formed at 20 °C show lower impedance values. Thus, anodizing in citric acid produces layers with remarkable barrier properties, particularly at lower temperatures.

Significant barrier ability is also observed in the layers obtained by anodization in tartaric acid (Figure 9c). In this case, the largest plot corresponds to the sample treated at 0 °C, exceeding the spectra of the samples treated at 20 °C. The shapes of the plots for these layers (i.e., those formed in tartaric acid electrolyte) resemble those of the films formed in citric acid, as discussed above.

Finally, the Nyquist plots of the films formed in glycine (Figure 9d) show lower impedance values than those of the reference samples, indicating deterioration of the native oxide layers on the AA2024-T3 surfaces, as discussed above.

In brief, the analysis of the Nyquist plots reveals the same trends as those summarized for the Bode plots. Accordingly, the layers derived from citric and tartaric acids possess notable barrier properties, unlike those obtained in oxalic acid. Lower process temperature leads to the formation of layers with slightly enhanced barrier properties. In contrast, glycine adversely affects the overall surface properties of the metallic substrates.

**Figure 10:**
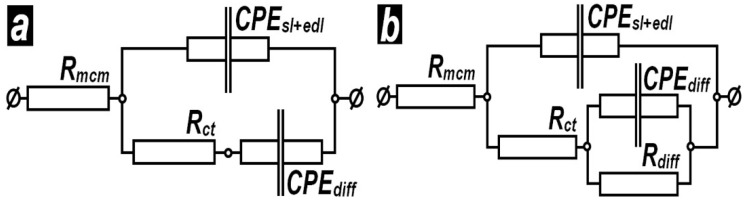
Model equivalent circuits (MECs) used for impedance spectra fitting for the references and the samples with inferior (**a**) and with superior (**b**) barrier ability.

Subsequently, the EIS spectra were subjected to modeling using suitable model equivalent circuits (MECs), as depicted in Figure 10. This approach enables differentiation of the barrier properties of the obtained layers, as well as characterization of the interfaces within the metal/surface layer/model corrosive medium system, as described elsewhere [162,163]. The MECs used in the present study consist of the resistance of the model corrosive medium (*R*_mcm_) and a constant phase element (*CPE*_sl+edl_), which describes the dielectric properties of the electrochemically formed solid layers (*sl*) together with those of the electric double layer (*edl*) at the interface between the model corrosive medium and the sample surface.

The *R*_ct_ element is attributed to resistance to charge-transfer reactions that constitute the corrosion process. Finally, the diffusion of species in the model corrosive medium (e.g., H_3_O^+^, OH^−^, Cl^−^ ions, and dissolved oxygen) is represented by two elements: *CPE*_diff_ and *R*_diff_, respectively.

The data obtained from fitting the EIS spectra shown in Figure 8 and Figure 9 using the model equivalent circuits presented in Figure 10 are summarized in Table 3.

The resistance of the model corrosive medium (*R*_mcm_) is relatively high, at about 500–600 Ω cm^2^, due to the low NaCl concentration, as noted in the experimental part of the present study. The *CPE*_sl+edl_ values resemble those of capacitors, with C ≈ 100–160 μF cm^−2^ for the reference samples. These values decrease by about three times in the case of oxalic acid, whereas for glycine, they are approximately twice as high. These findings indicate that anodization in oxalic acid results in clearly porous layers, whereas glycine causes deterioration of the native oxide layer. Together with the reference ones, the layers formed in these acids are considered as possessors of inferior barrier ability. Their spectra were fitted to the MEC, shown in Figure 10a. Since it does not contain *R_diff_* element and the absences of its values are marked by dashed lines in Table 3.

In the cases of citric and tartaric acids, the *CPE*_sl+edl_ values are five orders of magnitude lower for the layers formed at 0 °C and about four orders of magnitude lower for the films obtained at 20 °C. The exponential multiplier (n) takes values close to 0.80 in all cases. This indicates that *CPE*_sl+edl_ does not behave as an ideal capacitor, due to defects in the layers, including those in the native oxides of the reference samples.

The charge-transfer resistance (R_ct_) values of the reference samples are close to *R*_ct_ ≈ 5 kΩ cm^2^. Again, anodizing in oxalic acid results in almost twice the *R*_ct_, whereas treatment in a glycine electrolyte yields values even lower than those of the references.

In the case of citric acid, the *R*_ct_ values possess an order of magnitude higher values after treatment at 0 °C, whereas at 20 °C, these values are even lower than those of the reference samples. However, these *R*_ct_ values are compensated by an additional *R*_diff_ element, which has incomparably higher values, exceeding those of *R*_ct_ by two to three orders of magnitude. Additionally, the *R*_diff_ values of the samples treated at the lower temperature are about one order of magnitude higher than those of the samples anodically polarized at 20 °C.

The trends observed in the R_ct_ and R_diff_ values for the layers formed in tartaric acid are similar to those observed in citric acid, as noted above.

In summary, the AAO layers formed in citric and tartaric acid possess better barrier properties than those obtained in oxalic acid and glycine.

#### 3.5.2. Potentiodynamic Polarization Scanning

**Figure 11:**
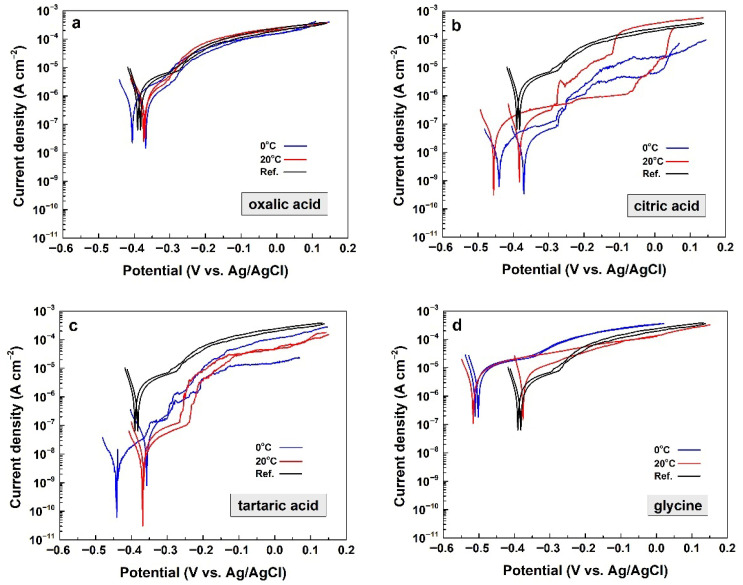
Juxtaposition among Potentiodynamic polarization curves were acquired from the studied specimens, including the reference samples for: (**a**) oxalic acid, (**b**) citric acid, (**c**) tartaric acid and (**d**) glycine.

This method, also known as Linear Sweep Voltammetry, was used to confirm the results obtained from the EIS spectra. All potentiodynamic scans are summarized in Figure 11.

The combination of all curves in Figure 11 enables a comparative analysis. It shows that the potentiodynamic curves for oxalic acid (Figure 11a) overlap those of the reference samples. This indicates that anodic polarization in this acid does not lead to the formation of layers with distinguishable barrier properties, as discussed above, due to their porosity.

The curves for the citric and tartaric acids (Figure 11b,c) are below those of the reference samples. This indicates that the current densities through the samples are lower, due to the apparent barrier properties of the electrochemically formed layers in citric and tartaric acids.

Finally, the potentiodynamic curves of the samples that underwent electrochemical treatment in glycine lie higher than those of the reference samples (Figure 11d). Only the curve of sample 1, treated at 20 °C, is close to those of the references. Nevertheless, this exception does not contradict the general inference that anodic polarization in glycine is detrimental. In other words, anodic polarization in this acid does not form a protective layer but rather dissolves the native oxide layer on the AA2024-T3 substrate.

The potentiodynamic curves were further analyzed using the Tafel slope to obtain numerical data on the barrier properties of the studied layers. For comparison, the curves of the reference samples were also analyzed. The resulting numerical data are summarized in Table 4.

The polarization resistance (*R*_p_) of the native oxide layers on the bare AA2024-T3 alloy (i.e., the reference samples) ranges from *R*_p_ ≈ 6 to 8 kΩ cm^2^, corresponding to corrosion current densities between 1 and 2 μA cm^−2^. These values are used to compare the barrier properties of the obtained layers, as shown in Table 4.

The layers electrochemically formed at 0 °C show significant differences depending on the organic acid electrolyte used. The layers formed in citric and tartaric acids possess significantly higher *R*_p_ values, compared to those of the other sample couples. This indicates that the respective layers exhibit well-developed barrier properties. However, large deviations are observed between samples within each couple, revealing relatively low repeatability. The *R*_p_ values of the layers obtained in oxalic acid are approximately twice those of the references. Consequently, the layers obtained in oxalic acid at 0 °C possess weak barrier properties, which become even weaker at 20 °C.

Finally, the respective *R*_p_ values recorded for the layers formed in the glycine indicate that this electrolyte has a detrimental effect, due to the dissolution of the native oxide layer on the AA2024-T3 alloy.

The further analysis has revealed an even alteration of the corrosion mechanism, depending on the organic acid used for anodization. Thus, the references and the layers formed in glycine underwent uniform corrosion, as appointed in Table 4. 

Localized (i.e., pitting) corrosion appears in the cases of oxalic, citric and tartaric acids. The pitting potentials (*E*_pit_) possess values around −300 mV versus the reference electrode (RE), similar to the corrosion potentials (*E*_corr_). These variations in both potentials predetermine differences in the strength against pitting nucleation (SAPN). The SAPN values are determined as the difference between *E*_pit_ and *E*_corr_. This difference reflects the width of the passivation region between *E*_pit_ and *E*_corr_. The highest resistance to pitting nucleation is observed for the samples prepared in tartaric acid, followed by those formed in citric and oxalic acids.

The comparison of the numerical data for the samples anodized at 0 °C and 20 °C shows that the effect of the process temperature is weaker than that of the electrolyte composition.

In conclusion, a good concordance is observed between the results obtained by the electrochemical analytical methods used, namely, electrochemical impedance spectroscopy and linear sweep voltammetry. According to both methods, citric and tartaric acids yield layers with notable barrier properties. In contrast, oxalic acid and glycine-based electrolytes yield layers with negligible barrier properties or deteriorate the protective properties of the native oxide layer.

## 4. Conceptual Summary

The variety of analytical techniques employed in the present study has provided extensive data regarding the properties, structure and composition of the obtained layers. The first notable difference among the layers formed in the respective organic acids is observed in their *U*_f_(*t*) kinetic curves. As discussed in Section 3.1, anodization in citric and tartaric acids reaches voltage values of up to approximately 400 V in order to maintain the applied current density of 15 mA cm^−2^. These high values are accompanied by oscillations, indicating layer breakdown followed by rapid re-passivation, also evinced by the respective SEM images in Figure 4. These features suggest that the layers formed in citric and tartaric acids are dense. Further chemical analyses (Section 3.4.3) have shown that these layers are composed of Al_2_O_3_, with small amounts of AlO(OH), along with traces of complex structures and bicarbonate species.

The subsequent corrosion tests, described in Section 3.5, revealed that these layers possess higher barrier properties, compared to those formed in oxalic acid and glycine. The following inferences can be drawn based on the respective resistance values (*R*_ct_ and *R*_diff_ from EIS, combined with *R*_p_ and *R*_pit_ from the polarization curve analysis).

According to the sum of *R*_ct_ and *R*_diff_, the barrier abilities can be ordered as follows:

*R*_ct_ + *R*_diff_ = 4.25 MΩ cm^2^ for tartaric acid at 0 °C > 3.02 MΩ cm^2^ for citric acid at 0 °C > 1.36 MΩ cm^2^ for tartaric acid at 20 °C > 0.61 MΩ cm^2^ for citric acid at 20 °C.

According to the sum of *R*_p_ and *R*_pit_, recorded for the best-performing samples of the respective sets, the barrier abilities can be ordered as follows:

*R*_p_ + *R*_pit_ = 10.96 MΩ cm^2^ for tartaric acid at 0 °C > 3.1 MΩ cm^2^ for citric acid at 0 °C > 2.61 MΩ cm^2^ for tartaric acid at 20 °C > 0.72 MΩ cm^2^ for citric acid at 20 °C.

Thus, this additional analysis of the corrosion test results, presented in Section 3.5, Table 3 and Table 4, reveals (i) satisfactory agreement between the results obtained by EIS and potentiodynamic polarization measurements; (ii) superior barrier properties of the layers formed in tartaric acid compared to those obtained in citric acid; and (iii) a better protective effect of the layers formed at the lower temperature (i.e., 0 °C compared to 20 °C).

This ordering of the barrier properties should be related to other characteristics, such as wettability, layer thickness, and density. The wettability tests (Section 3.4.2) show that according to the contact angle (CA), the ordering from less hydrophilic to more hydrophilic is as follows: CA (citric acid, 20 °C) > CA (citric acid, 0 °C) > CA (tartaric acid, 20 °C) > CA (tartaric acid, 0 °C). Thus, the more hydrophilic layers appear to exhibit higher barrier properties. This observation contradicts the commonly accepted correlation between hydrophobicity and barrier properties, which is typically attributed to the repulsion of the corrosive medium from the surface.

**Figure 12:**
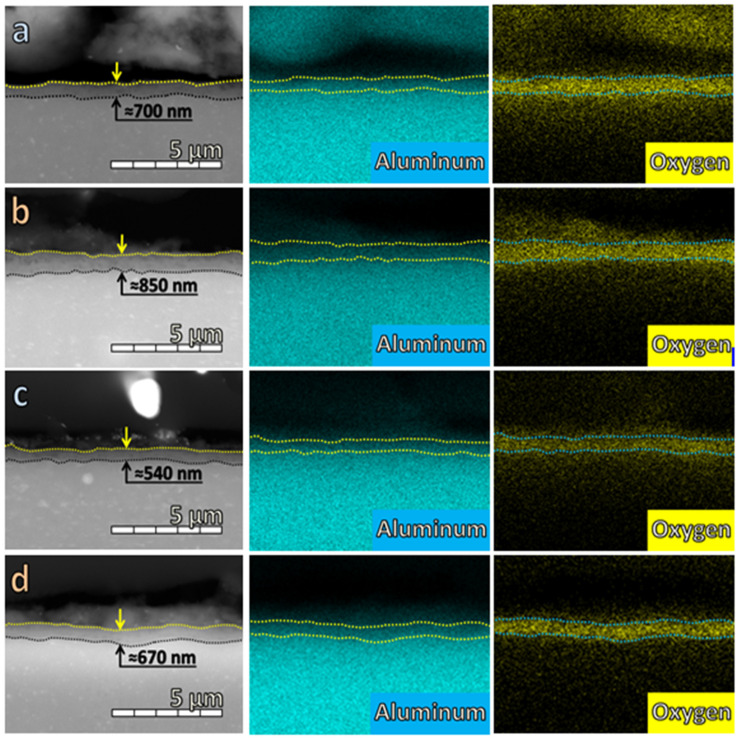
Cross-sectional SEM images, combined with EDX maps for citric acid at 0 °C (**a**) and 20 °C (**b**), and tartaric acid at 0 °C (**c**) and at 20 °C (**d**).

This apparent contradiction necessitated additional cross-sectional SEM image analysis in order to determine whether the observed ordering of barrier properties of the layers is related to their thickness. The corresponding cross-sectional SEM images, combined with EDX mapping analyses, are presented in Figure 12.

The comparison of the layer thicknesses (*τ*) reveals the following order: *τ* (citric acid, 20 °C) > *τ* (citric acid, 0 °C) > *τ* (tartaric acid, 20 °C) > *τ* (tartaric acid, 0 °C). This order is fully consistent with that of the contact angle. In other words, the thinner layers possess more hydrophilic surfaces.

However, the highest barrier properties are observed for the most hydrophilic layer, which is also the thinnest one (i.e., the layer formed by anodization in tartaric acid at 0 °C). Thus, both the hydrophobicity and thickness trends are inversely proportional to the trend of the barrier ability, as established by the corrosion tests.

This observation suggests that, at the initial stage of exposure of the layers to the model corrosive medium, the thinner and more hydrophilic ones more readily absorb its components (e.g., H_3_O^+^, OH^−^, Cl^−^ ions, H_2_O molecules, dissolved oxygen, etc.). These species promote dissolution of Al from the underlying metallic substrate across defects in the layer. The resulting Al^3+^ ions react with the above-mentioned species, forming insoluble products, such as hydrated Al(OH)_3_, boehmite, and related compounds, including Keggin-type clusters/polyoxoaluminates, as reported elsewhere [1,164,165,166,167]. In turn, these insoluble products hinder further penetration of corrosive species, thereby forming an effective barrier against subsequent corrosion propagation. In addition, swelling of superficial [(M^II^)_8_(C_4_O_7_)_4_(H_2_O)_12_]_24_·H_2_O species and the formation of complexes with Al^3+^ ions probably also contribute to restricting the access of corrosive species to the metallic surface beneath the layer defects.

It should also be noted that additional experiments were performed in 3.5% NaCl solution. The results indicate that anodization in these organic acids does not provide layers with barrier properties comparable to those formed in sulfuric acid, as reported elsewhere [168,169]. Therefore, it appears more appropriate to use these acids as additives to sulfuric acid electrolytes, as proposed in previous studies on citric-sulfuric [170,171,172], tartaric-sulfuric [173,174,175,176,177,178] and succinic-sulfuric [172] systems, including additional ceria-based sealing [178,179]. In this context, the organic anions, such as oxalate, citrate, and tartrate, are capable of forming chelate complexes that can hinder the access of corrosive species to the protected metal surface, as discussed in recent studies [180,181,182,183,184,185].

## 5. Conclusions

The effects of four environmentally conscious organic acid electrolytes on the anodic polarization of AA2024-T3 alloy were characterized. The experiments were conducted in oxalic, citric, tartaric acid, and glycine electrolytes of the same molarity at two temperatures (i.e., 0 and 20 °C). The comparative analysis also included reference samples of the bare alloy. The investigations encompassed film formation kinetics and the resulting layer characteristics. Thus, the topologies, surface properties (color characteristics and wettability), chemical composition, and corrosion resistance of the obtained layers were analyzed.

The analysis of the acquired data has led to the conclusion that, according to their protective ability, the obtained layers can be ordered as follows: tartaric acid at 0 °C > citric acid at 0 °C > tartaric acid at 20 °C > 0 citric acid at 20 °C.

This trend does not coincide with either the hydrophobicity or the thickness order. Surprisingly, the thinnest and most hydrophilic layer (formed at 0 °C in tartaric acid) exhibits the highest barrier ability.

This discrepancy is explained by the concept proposed and discussed in the conceptual summary of the present study.

Finally, although the investigated organic acids and anodizing regimes resulted in AAO layers with distinguishable barrier properties, these layers are not able to substitute classical H_2_SO_4_-derived layers. Nevertheless, the present comparative study provides insight into the potential use of these acids as additives to sulfuric acid-based electrolytes, as well as the expected effects of their application.

## Figures and Tables

**Table 1:** Color characteristics of the investigated samples after anodizing at 0 and 20 °C.

Temperature	Electrolyte	L*	a*	b*
0 °C	Oxalic acid	65.95 ± 2.37	2.49 ± 0.46	2.95 ± 0.64
	Citric acid	93.24 ± 8.27	0.93 ± 0.37	10.89 ± 1.75
	Tartaric acid	89.96 ± 2.00	−2.63 ± 0.89	1.56 ± 0.73
	Glycine	92.40 ± 5.61	0.83 ± 0.48	7.53 ± 1.57
20 °C	Oxalic acid	90.35 ± 9.12	3.00 ± 2.13	10.60 ± 5.17
	Citric acid	94.20 ± 8.67	0.91 ± 0.43	11.26 ± 2.26
	Tartaric acid	90.28 ± 0.72	0.40 ± 0.43	4.51 ± 0.21
	Glycine	96.27 ± 5.55	0.51 ± 1.07	4.37 ± 4.18

**Table 2:** Contact angles of the investigated samples after anodization at 0 and 20 °C.

Temperature	0 °C	20 °C
Electrolyte	Contact Angle [°]	Contact Angle [°]
Oxalic acid	25.97 ± 7.67	26.84 ± 3.43
Citric acid	75.88 ± 8.27	80.86 ± 11.86
Tartaric acid	47.81 ± 18.03	67.17 ± 15.15
Glycine	40.48 ± 12.76	45.83 ± 11.44

**Table 3:** Results of EIS spectra fitting using suitable MECs for the investigated specimens, including the reference samples.

Sample		*R*_mcm,_ (Ω cm^2^)	*CPE*_sl+edl_ (s^n^ Ω^−1^ cm^−2^) × 10^−6^	*n* (/)	*R*_ct_ (kΩ cm^2^)	*CPE*_diff_ (s^n^ Ω^−1^ cm^−2^) × 10^−6^	*n* (/)	*R*_diff_ (MΩ cm^2^)
**Reference**	**S_1_** **S_2_**	620.0 ± 3.8 556.0 ± 3.1	153.80 ± 8.31 116.00 ± 5.49	0.80 ± 0.01 0.81 ± 0.01	4.15 ± 0.51 5.64 ± 0.79	399.20 ± 26.27 300.50 ± 32.37	0.58 ± 0.01 0.53 ± 0.01	----------- -----------
**Oxalic acid** **0 °C**	**S_1_** **S_2_**	554.0 ± 71.0 510.2 ± 18.4	50.92 ± 1.33 81.74 ± 2.16	0.80 ± 0.01 0.80 ± 0.01	10.41 ± 0.02 10.43 ± 0.02	35.08 ± 1.26 36.73 ± 1.94	0.56 ± 0.01 0.58 ± 0.01	----------- -----------
**Oxalic acid** **20 °C**	**S_1_** **S_2_**	604.0 ± 7.0 633.0 ± 6.0	64.33 ± 7.0 83.50 ± 4.2	0.79 ± 0.01 0.80 ± 0.01	11.91 ± 3.60 10.46 ± 1.53	160.20 ± 40.27 286.20 ± 58.43	0.55 ± 0.01 0.53 ± 0.01	----------- -----------
**Citric acid** **0 °C**	**S_1_** **S_2_**	572.0 ± 17.9 548.0 ± 59.0	(75.61 ± 2.16) × 10^−3^ (62.74 ± 16.65) × 10^−3^	0.89 ± 0.01 0.91 ± 0.01	44.80 ± 3.51 34.23 ± 1.46	(56.63 ± 0.82) × 10^−2^ (51.47 ± 0.15) × 10^−2^	0.61 ± 0.01 0.65 ± 0.01	2.98 ± 0.04 1.75 ± 0.09
**Citric acid** **20 °C**	**S_1_** **S_2_**	532.0 ± 56.0 565.0 ± 51.4	(12.16 ± 4.09) × 10^−2^ (23.09 ± 6.60) × 10^−2^	0.85 ± 0.01 0.79 ± 0.01	4.75 ± 0.36 2.23 ± 0.10	3.28 ± 0.07 9.95 ± 0.13	0.65 ± 0.01 0.70 ± 0.01	0.61 ± 0.02 0.57 ± 0.02
**Tartaric acid** **0 °C**	**S_1_** **S_2_**	561.0 ± 27.7 501.4 ± 31.5	(91.78 ± 29.9) × 10^−3^ (99.30 ± 0.24) × 10^−3^	0.92 ± 0.01 0.57 ± 0.01	17.24 ± 9.76 27.47 ± 9.51	(58.30 ± 0.82) × 10^−2^ (14.82 ± 11.80) × 10^−2^	0.59 ± 0.01 1.00 ± 0.01	4.24 ± 0.20 0.49 ± 0.04
**Tartaric acid** **20 °C**	**S_1_** **S_2_**	591.0 ± 32.3 578.0 ± 23.5	(20.24 ± 6.06) × 10^−2^ (14.80 ± 1.61) × 10^−2^	0.86 ± 0.01 0.84 ± 0.01	5.12 ± 0.56 69.10 ± 16.56	3.52 ± 0.09 0.43 ± 0.01	0.63 ± 0.01 0.61 ± 0.01	1.36 ± 0.08 1.02 ± 0.07
**Glycine** **0 °C**	**S_1_** **S_2_**	568.0 ± 2.0 623.0 ± 1.5	320.5 ± 20.5 261.5 ± 12.0	0.59 ± 0.01 0.60 ± 0.01	1.10 ± 0.08 0.96 ± 0.04	1172.00 ± 26.48 1175.00 ± 26.48	0.69 ± 0.01 0.70 ± 0.01	----------- -----------
**Glycine** **20 °C**	**S_1_** **S_2_**	555.0 ± 1.5 583.0 ± 1.2	135.60 ± 7.18 198.73 ± 2.44	0.74 ± 0.01 0.74 ± 0.01	0.73 ± 0.02 1.49 ± 0.03	1056.00 ± 9.33 1153.00 ± 16.02	0.73 ± 0.01 0.58 ± 0.01	----------- -----------

**Table 4:** Results of the Tafel slope analysis for the studied specimens, including the reference samples.

Electrolyte	*T*	Sample	OCP (mV)	*E*_corr_ (mV)	*R*_p_ (kΩ cm^2^)	*E*_pit_ (mV)	SAPN	*R*_pit_ (kΩ cm^2^)
**References**		**S_1_**	−368	−390	6.639	Uniform corrosion		
		**S_2_**	−362	−382	8.329			
**Oxalic acid**	**0 °C**	**S_1_**	−353	−368	21.840	−297	71	96.71
		**S_2_**	−392	−406	17.690	−321	85	83.13
	**20 °C**	**S_1_**	−360	−373	17.78	−306	67	85.21
		**S_2_**	−359	−370	12.88	−311	59	98.23
**Citric acid**	**0 °C**	**S_1_**	−431	−442	1.1 × 10^3^	−279	163	2.00 × 10^3^
		**S_2_**	−355	−370	785.00	−274	96	3.17 × 10^3^
	**20 °C**	**S_1_**	−443	−456	252.00	−62	394	395.95
		**S_2_**	−364	−383	198.60	−278	105	522.20
**Tartaric acid**	**0 °C**	**S_1_**	−431	−441	2.1 × 10^3^	−363	78	8.86 × 10^3^
		**S_2_**	−353	−357	247.20	−299	58	1.22 × 10^3^
	**20 °C**	**S_1_**	−350	−367	564.20	−266	101	2.05 × 10^3^
		**S_2_**	−357	−369	1.1 × 10^3^	−238	131	1.80 × 10^3^
**Glycine**	**0 °C**	**S_1_**	−488	−511	2.192			
		**S_2_**	−392	−406	2.248	Uniform corrosion		
	**20 °C**	**S_1_**	−350	−376	2.369			
		**S_2_**	−499	−516	3.202			

## Data Availability

The original contributions presented in this study are included in the article. Further inquiries can be directed to the corresponding author.
